# Assessment of central pain sensitization in chronic low back pain and its utility in diagnosis and prediction of clinical severity

**DOI:** 10.1038/s41598-025-33514-5

**Published:** 2026-01-07

**Authors:** Pablo de la Coba, Ailyn Garcia-Hernandez, Pilar Aranda-Villalobos, Juan A. Andrade-Ortega, Gustavo A. Reyes del Paso

**Affiliations:** 1 Department of Psychology and Anthropology, University of Extremadura, Cáceres & Badajoz, Spain; 2 Department of Rehabilitation, Complejo Hospitalario de Jaén, Jaén, Spain; 3 Department of Psychology, University of Jaén, Jaén, Spain

**Keywords:** Central sensitization, Chronic low back pain, Evoked pain, Temporal summation

## Abstract

Slowly repeated evoked pain (SREP) is a dynamic pain indicator useful in conditions involving central sensitization. This study evaluated SREP in patients with chronic low back pain (CLBP) and compared it to temporal summation of pain (TSP). The SREP protocol involved nine low-to-moderate painful pressure stimuli (5 s each, 30-s intervals) in 40 patients with CLBP and 40 healthy individuals. Clinical-psychological factors, pain threshold, tolerance, and TSP were assessed. Patients with CLBP showed increasing pain ratings to SREP (0.78 ± 1.05), while healthy individuals did not (−0.44 ± 1.10). No significant group differences were found in TSP, pain threshold and tolerance. SREP effectively distinguished patients with CLBP from healthy individuals (66.3% accuracy), with an optimal cut-off of 0.05 (sensitivity = 77.5%, specificity = 60%), while other pain tests did not. Usual and current pain, central sensitization-related symptoms, and catastrophizing correlated with TSP but not SREP. Sensitization to SREP distinguished patients with CLBP from healthy individuals while TSP did not provide discriminatory ability. However, SREP did not predict clinical severity of the patients, whereas TSP allowed for the prediction of clinical severity. Future development in the application of SREP protocol could help detect and assess the centralized pain component in CLBP.

## Introduction

Low back pain, characterized by localized pain below the costal margin and above the inferior gluteal folds, with or without referred leg pain, has a lifetime prevalence exceeding 80% in high-income countries^1^. Globally, low back pain is the most common musculoskeletal condition and a leading cause of disability, representing a serious social and economic challenge worldwide^1–3^. According to the International Association for the Study of Pain, when the pain in the lower back lasts or recurs for more than 3 months, is linked to emotional distress or functional impairment, and cannot be fully explained by another identifiable cause, such as an injury, structural damage, or an underlying disease, is defined as chronic low back pain (CLBP)^4^.

The diagnosis of CLBP is based on clinical examination, but there is no single or well-established method for the diagnosis of these patients^5,6^. Likewise, there is no single cause of this chronic pain condition, but there are multiple possible generators of pain that could differentiate the cause of CLBP as predominantly nociceptive, neuropathic or centralized^7,8^. Additionally, pain sensitization processes (at the central and peripheral levels) seem to be key factors in the chronification of low back pain^1^, where central sensitization (CS) to pain is seen in a considerable proportion of patients with CLBP (nearly 50%)^8,9^.

The concept of CS is understood as an increase in pain responsiveness due to altered nociception in the central nervous system (at the brain and/or spinal cord level) that can be explained by long-term potentiation, upregulation of facilitatory ascending pain pathways, and/or dysfunctional descending pain inhibition^10^. However, while CS can be measured in animals via invasive techniques, it is “simply” estimated or assumed in humans^8,11^. Non-invasive or indirect CS measures include the use of (I) neuroimaging^12^, (II) questionnaires to assess CS-related symptoms (e.g., the Central Sensitization Inventory, CSI)^13^, and (III) algometry measures including different evoked pain indicators [e.g., temporal summation of pain (TSP) or conditioned pain modulation (CPM)]. These indicators are often included within broader assessment protocols such as quantitative sensory testing (QST)^14^. Algometry measures are readily available, quick to use, and simpler than neuroimaging techniques. Although they are semi-subjective measures (relying on the participant’s introspection), they could be preferable to questionnaires, which only assess the frequency of CS-related symptoms (partly associated with CS) and not the change in responsiveness due to CS processes.

The assessment of pain sensitivity through static evoked pain measures (e.g., pain threshold or tolerance) and of pain responsiveness through dynamic pain indicators (e.g., TSP, based on the spinal wind-up effect^15^, or CPM, based on descending pain inhibition^16^) has been extensively investigated. Static measures consistently reveal greater pain sensitivity (reflected by lower pain threshold and tolerance) in patients with CLBP compared with pain-free individuals^17–19^. Meanwhile, dynamic indicators are thought to be more specifically sensitive to CS^20,21^. While several studies report an enhanced TSP response^18,19^ and reduced CPM^17^ efficiency in patients with CLBP relative to controls, other evidence suggests that these measures have only limited discriminative value, showing small effect sizes and inconsistent results across studies^22–24^.

In turn, clinical and psychological factors usually influence these evoked pain measures, becoming key factors for interpreting pain responses and differentiating CLBP profiles^25^. Pain catastrophizing, anxiety, fear of pain, sleep problems, and depression are among the factors with proven effects on pain sensitivity and responsiveness in both pain-free^26,27^ and CLBP individuals^28,29^. Furthermore, current pain intensity and the degree of variation in clinical pain also modulate pain responses^30–32^.

Previous studies using a slowly repeated evoked pain (SREP) protocol have concluded that SREP is capable of discriminating between pain conditions that differ in CS. Enhanced pain sensitization as a response to the SREP protocol has been found in the majority (approximately 90%) of females with fibromyalgia^33–35^ and, to a lesser extent, in patients with episodic migraine^36^. However, SREP sensitization was not observed in free-pain individuals^33–36^ or patients with pain primarily related to peripheral sensitization, such as rheumatoid arthritis^37^. The ability to discriminate conditions differing in levels of CS by SREP was superior to that of the static evoked pain measures of pain threshold and tolerance. The accuracy indexes of SREP for discriminating patients with fibromyalgia from healthy or rheumatoid arthritis individuals were both > 85%, and between 70 and 75% for differentiating patients with migraine from healthy individuals; whereas the accuracy indexes of pain threshold and tolerance for discriminating patients with fibromyalgia from healthy or rheumatoid arthritis individuals were between 50 and 70%, and around 50% (as chance) for differentiating patients with migraine from healthy individuals^34,36,37^. In comparison to a well-established dynamic pain indicator such as TSP, SREP has also shown a significantly greater discrimination accuracy. The accuracy index of TSP for discriminating fibromyalgia from rheumatoid arthritis was < 65%, and around 50% for differentiating patients with migraine from healthy subjects (i.e., inability to distinguish between them)^36,37^. Moreover, SREP sensitization, again compared to TSP, has shown a greater test–retest reliability (0.80 vs. 0.68 in fibromyalgia and 0.73 vs. 0.49 in rheumatoid arthritis, for SREP vs. TSP, respectively) and appears to be more strongly associated with levels of clinical pain and other related clinical and psychological factors (e.g., fatigue or sleep satisfaction)^33–37^. Overall, the exploration of the utility of SREP protocol in other CS-related pain conditions, like CLBP, is justified, facilitating the development of this promising dynamic pain indicator. To our knowledge, this would be the first study to implement the SREP protocol in a cohort of patients with CLBP.

However, unlike other dynamic measures such as TSP or CPM, whose underlying neural mechanisms and pathways have been more extensively investigated, SREP still lacks direct evidence clarifying the neurophysiological substrates that drive its sensitization response^21^. Comparative studies between SREP and TSP suggest that, although both may share certain mechanisms of CS, SREP is likely to rely on additional, distinct processes. This interpretation is supported by the marked difference in stimulation frequency between the protocols: TSP effects are elicited at approximately 0.33 Hz, whereas SREP is observed at around 0.03 Hz, a rate that does not induce the wind-up phenomenon characteristic of TSP and may instead engage alternative inhibitory or modulatory pathways^21,37^.

Therefore, considering the large proportion of patients with CLBP whose pain seems to underlies CS processes^8,9,38^ and the utility of SREP to index pain sensitization in CS conditions, this study aimed to assess the usefulness of SREP in characterizing CS-related pain responsiveness among patients with CLBP. Thus, the following objectives were proposed: (1) to characterize the response to the SREP protocol of patients with CLBP, using TSP as the reference dynamic evoked pain measure; (2) to examine the associations between the severity of clinical-psychological symptoms and the SREP and TSP indicators; and (3) to evaluate the ability of SREP to discriminate between patients with CLBP and healthy individuals, in comparison with the most commonly used static (pain threshold and tolerance) and, in particular, dynamic (TSP) evoked pain measures. Accordingly, we formulated three corresponding hypotheses: (1) whereas TSP response would be greater in patients with CLBP than healthy individuals, SREP sensitization would be present in the patients, but blunted or absent in healthy individuals; (2) higher levels of SREP sensitization and a greater TSP response are predicted to be associated with more severe clinical symptoms; and (3) the discriminative capacity of the SREP index (based on the amplitude of the pain sensitization response) for differentiating between patients with CLBP and healthy individuals is expected to be greater than that of TSP and static pain measures.

## Materials and methods

### Eligibility criteria

There were three exclusion criteria for all participants: (I) being under 40 or over 65 years of age (it was attempted to limit the age range to a relatively narrow range, given the relationship between older age and higher levels of CS)^39,40^; (II) the presence of any severe medical pathology (e.g., cancer) or psychiatric disorder; and (III) a body mass index (BMI) ≥ 40, given that morbid obesity is known to involve musculoskeletal structural damage and a chronic pro-inflammatory state that may distort pain perception^41^.

Regarding the patients with CLBP, they specifically met the following inclusion criteria: (a) to have received a diagnosis of CLBP from rheumatologists in the public rehabilitation service of Hospital de Jaén, who adhered to the International Association for the Study of Pain guidelines for CLBP diagnosis^4^; and (b) a chronicity ≥ 1 year (estimated from the time at which the first back pain symptoms arose). Likewise, the following exclusion criterion was specifically established for the patients: suffering from any comorbid pain condition other than low back pain (e.g., rheumatoid arthritis). In relation to the healthy individuals, they specifically met the following exclusion criterion: suffering from any acute/chronic pain condition, or have suffered from chronic pain in any time of the past, which explicitly included any episode of low back pain.

### Recruitment

Patients with CLBP were recruited from the public rehabilitation service of the Hospital de Jaén. Recruitment was initiated once the diagnosis of CLBP had been confirmed by the attending rheumatologists. At that point, eligible patients were invited to participate in the study. Healthy individuals were recruited using a snowball sampling method, primarily through relatives of students at the University of Jaén. To ensure their integrity as a pain-free individuals, these were screened based on the exclusion criterion previously described.

### Participants

After applying the eligibility criteria and conducting the recruitment above explained, 40 patients with CLBP and 40 healthy individuals participated in this study. The patient sample consisted of 24 women and 16 men with CLBP (60% women) with a mean age of 52.90 ± 5.22 years, and the healthy sample comprised 20 women and 20 men with a mean age of 50.80 ± 5.91 years. There was no significant difference in age between patients and healthy individuals [*t*(39) = 1.69, *p* = 0.10]. Both samples were similar in terms of BMI (26.95 ± 3.48 kg/m^2^ and 25.78 ± 3.28 kg/m^2^, for patients with CLBP and healthy individuals, respectively, *t*(39) = 1.55, *p* = 0.13).

Relevant features of the sample of patients were: (1) the majority suffered from intervertebral disc alterations (e.g., herniation), facet joint problems, or spinal stenosis; (2) all of them met the criteria of recurrent low back pain (defined as the recurrence of low back pain within 12 months after recovery from their first episode)^42^; and (3) the average chronicity was 16.12 ± 14.40 years, which did not correlate with any evaluated variable (all ps ≥ 0.08).

### Clinical and psychological assessment

All participants were evaluated using the Spanish versions of following questionnaires, administered by a trained interviewer in a structured format. The assessment was conducted through face-to-face interviews that lasted approximately 30 min: (a) the Coping Strategies Questionnaire, which though includes 8 different subscales, only its catastrophizing subscale (CSQ-Cat)^43^ was used for the study. The CSQ-Cat is composed of six items with a score range of 0–36 and an internal consistency of α = 0.89^43^; (b) the Hospital Anxiety and Depression Scale (HADS)^44^. The HADS evaluates anxiety and depression in clinical populations; each subscale consists of seven items, with a score range of 0 to 21 and an internal consistency of α = 0.86^45^; (c) the State-Trait Anxiety Inventory, specifically the State subscale (STAI-S)^46^. This scale evaluates current anxiety level through 20 Likert scale items, with a score range of 0–60 and an internal consistency of α = 0.93^46^; (d) the sleep satisfaction subscale of the Oviedo Sleep Questionnaire (COS-Sat)^47^. This scale measures sleep satisfaction using a unique 7-point Likert scale ranging from 1 (Very Unsatisfied) to 7 (Very satisfied); and (e) a 0–10 visual analog scale (VAS) to quantify the intensity of current clinical pain.

Additionally, to evaluate the clinical severity of CLBP, the Spanish versions of the following questionnaires were administered to patients: (f) the McGill Pain Questionnaire (MPQ)^48^, that provides a total score based on sensory, emotional, miscellaneous and evaluative pain dimensions. Scores range from 0 to 66 based on word counting^48,49^; and (g) the CSI^50^, that measures the frequency of symptoms related to CS via 25 Likert scale items ranging from 0 (Never) to 4 (Always), resulting in a range score of 0 to 100. It has an internal consistency of α = 0.87^50^. Scores > 40 indicate CS^13^.

### SREP protocol

Pressure was applied by an algometer (Tracker Freedom; JTECH Medical, Lawndale, CA, USA) with a stimulation surface area of 1 cm^2^ and inserted in a piston to reliably maintain stimulation pressure. In the first step, pain threshold and tolerance were explained as “the lowest pressure stimulation that causes pain” (explained to participants as “the initial sensation of discomfort specifically resulting from a slight finger pain, a minor yet noticeable pain that is easily bearable but still painful”) and “the highest pressure stimulation that you are able to tolerate”, respectively. According to the described concepts of these evoked pain measures, necessary to apply later the SREP protocol, participants were instructed to respond verbally to indicate in the shortest possible time the moment at which minimal discomfort (pain threshold) or pain close to “unbearable” (pain tolerance) occurred, at which point pressure stimulation immediately ceased.

As in all previous SREP studies, stimulation with pressure increasing by 1 kg/s was delivered to the third fingernail of the left hand (non-dominant hand for all participants) for this study as well, given that the pressure on the fingernails reflects overall pain sensitivity^51^. To ensure greater reliability, both pain measures were obtained three times at 30-s intervals; the average of the three measurements was taken for the analysis^52^. A 1-min interval was left between the measurement of pain threshold and the measurement of pain tolerance. These two evoked pain measures were used to estimate the pain pressure for the SREP protocol (individually for each participant) as follows: Intensity = Threshold + 1.25*(DF/4); where DF = Tolerance−Threshold^53^.

Over a 3-min rest period, the VAS pain assessment procedure was practiced through a series of three stimuli, 5 s in duration and of different intensities (1.5, 2.5, and 2.0 kg/cm^2^ in sequence), applied to the second fingernail of the left hand.

Then, the SREP protocol was performed as a single series of nine low-to-moderate painful stimuli (as calculated above) applied to the third fingernail of the left hand for 5 s, followed by a 5-s waiting period, and 20 s during which the pain rating was recorded, with silence maintained immediately until the next trial. Resulting in an interval between stimuli of 30 s. Pain intensity for each painful stimulus was evaluated using a 10-cm VAS ranging from “No Pain” to “Extremely Painful”, which was presented to the participant justly after the 5 s of waiting period in each painful stimulus.

When the patient reported a pain rating, the researcher recorded the corresponding numerical score that appeared on the back of the VAS. The precision of the scale (and therefore of the SREP values) was to one decimal place. As a result, the total protocol duration was 4.5 min.

The SREP index was calculated as the difference between the ninth and the first VAS pain ratings (i.e., VAS rating 9 minus VAS rating 1), both obtained from the sequence of nine pressure stimulations. This index reflects the change in perceived pain across repeated stimuli, with higher positive values indicating greater sensitization or negative values point to a habituation of pain response to SREP.

### TSP protocol

The TSP protocol initially described by Goodin et al.^54^ to assess CS in knee osteoarthritis patients, and after utilized successfully in SREP vs. TSP studies in fibromyalgia, rheumatoid arthritis, episodic migraine and pain-free individuals^33–37^, was used to evoke TSP in this study.

First, similarly to SREP, the NRS pain rating procedure was practiced on the right hand to ensure participants had the ability to provide a verbal pain rating once per second (1 Hz) during a series of 10 punctuate stimuli. Afterwards, two TSP series of 10 punctuate stimuli were delivered at a rate of 1 Hz (1 stimulus/second) to the thenar eminence of the left hand, with an inter-series interval (rest) of 30 s. A nylon monofilament (Touchtest Sensory Evaluator 6.65) calibrated to bend at 300 gr of pressure was used. Pain ratings for each stimulus were acquired verbally through a 0–10-point numeric rating scale, with “0” indicating “no pain” and “10” indicating an “extremely painful” stimulus. The monofilament was pressed perpendicularly against the hand until it was bent at a 90º angle. Due to the inability to record the numerical values verbally reported by the participants, voice recordings were made during the two TSP series. Based on the average of the two TSP series, the TSP index was derived as the difference between the 10^th^ and the 1^st^ pain ratings (larger positive values indicate greater TSP).

### Procedure

The study was performed in a single session of approximately one hour, divided into three parts: (A) *clinical history and questionnaire assessment*, during which a psychologist checked the eligibility criteria in a semi-structured interview addressing sociodemographic data, clinical features, and regular medication use (non-opioid analgesics, opiates, anxiolytics, and antidepressants); questionnaires were then administered and participants’ weight and height were measured; (B) *SREP protocol*; and (C) *TSP protocol*, both described in the corresponding subsections above.

A 5-min rest period was implemented between the counterbalanced SREP and TSP protocols. Patients with CLBP and healthy individuals were evaluated in an alternating order, i.e., participant type was also counterbalanced. In both SREP and TSP protocols the participants were asked to look straight ahead to avoid seeing how and when each stimulus was applied. Participants were asked not to consume alcohol or caffeine, and not to engage in intense physical exercise for 24 h before the study. All participants were previously informed about the study protocol and provided written informed consent. The Ethics Committee of the University of Jaén approved the study (reference: FEB.23/2.PRY), which was performed in line with the updated Declaration of Helsinki^55^.

### Statistical analyses

To estimate sample size, the G*Power 3.1.7 program was used^56^. We based the calculations on previous SREP vs. other evoked pain indicators studies^33–36^, which showed large effect sizes in ANOVA models (both univariate and repeated measures) and linear multiple regression analyses (F of 0.40 and f^2^ of 0.35). Considering these data, and α = 0.05, a total sample size of n > 51 was found to be necessary for a power of 0.80 in ANOVAs, whereas a sample size of n > 35 per group was found to be sufficient to achieve the same power in regression analyses. Thus, according to these estimates, and given that the correlation analyses were conducted in the patient group, a sample size of n = 40 for each group was established. The Kolmogorov–Smirnov test showed no deviation from normality in the measured variables (*p* > 0.05), except for TSP-related variables (Z = 2.24, *p* < 0.001 for TSP; and Z = 1.81, *p* = 0.003 for the first TSP pain rating). Considering the TSP data distribution and unsuccessful attempts to normalize the distribution, non-parametric tests were conducted for this variable.

As a preliminary analysis to characterize the study samples, student’s t-tests for independent samples (Mann–Whitney U test for TSP-related variables) were performed to compare sociodemographic, clinical and evoked pain data between the groups (Table 1). The independent variable was Group (CLBP vs. healthy), and the dependent variables were sociodemographic, clinical, and evoked pain measures.

**Table 1:** Clinical and evoked pain data in patients with chronic low back pain (CLBP) vs. healthy participants.

Variables	Patients with CLBP (n = 40) Mean ± SD	Healthy participants (n = 40) Mean ± SD	t, U or χ^2^	*p*	η^2^, PS or φ_c_
Catastrophizing (CSQ-Cat)	10.85 ± 6.77	3.05 ± 5.59	5.62	< 0.001	0.29
State anxiety (STAI-S)	16.98 ± 11.44	10.43 ± 5.08	3.31	0.002	0.12
Sleep satisfaction (OSQ-Sat)	3.63 ± 1.92	5.18 ± 1.78	− 3.75	< 0.001	0.15
Anxiety (HADS-A)	7.73 ± 5.02	5.43 ± 3.78	2.32	0.023	0.06
Depression (HADS-D)	4.30 ± 4.56	2.28 ± 2.41	2.48	0.016	0.07
Current pain intensity (0–10 VAS)	3.50 ± 2.39	0.60 ± 1.45	6.57	< 0.001	0.36
Pain threshold (Kg)	3.63 ± 2.11	3.72 ± 1.60	− 0.21	0.833	< 0.01
Pain tolerance (Kg)	9.46 ± 2.48	9.29 ± 2.43	0.30	0.760	< 0.01
Pressure SREP (Kg/cm^2^)^a^	5.45 ± 2.02	5.46 ± 1.54	− 0.02	0.984	< 0.01
1^st^ SREP pain rating (0–10 VAS)	3.16 ± 1.85	3.18 ± 2.25	− 0.03	0.974	< 0.01
SREP	0.78 ± 1.05	− 0.44 ± 1.10	5.07	< 0.001	0.25
1^st^ TSP pain rating (0–10 NRS)	0.50 (0.00–1.75)^b^	1.00 (0.00–1.50)^b^	731.5	0.493	0.46
TSP	0.00 (0.00–0.50)^b^	0.00 (0.00–0.50)^b^	742.0	0.555	0.46
Analgesics use, n (%)	29 (72.5%)	2 (5%)	38.39	< 0.001	0.48
Opiates use, n (%)	15 (37.5%)	0 (0%)	18.46	< 0.001	0.23
Anxiolytics use, n (%)	25 (62.5%)	1 (2.5%)	32.82	< 0.001	0.41
Antidepresants use, n (%)	9 (22.5%)	2 (5%)	5.17	0.023	0.06

Group means ± standard deviations, group comparisons (*t*, U or χ^2^, and *p*) and effect sizes (η^2^, PS or φ_c_).

Abbreviations. CLBP = chronic low back pain CSI = Central Sensitization Inventory; CSQ-Cat = pain catastrophizing subscale of Coping Strategies Questionnaire; HADS = Hospital Anxiety (A) and Depression (D) Scale; MPQ = McGill Pain Questionnaire; NA = not applicable; NRS = Numeric Rating Scale; OSQ-Sat = Satisfaction subscale of Oviedo Sleep Questionnaire; PS = probability of superiority; SREP = slowly repeated evoked pain sensitization, derived from the difference between 9th and 1st SREP pain rating; STAI-S = State subscale of State-Trait Anxiety Inventory; TSP = Temporal Summation of Pain response, derived from the average difference between 10th and 1st pain rating in the two TSP series; VAS = visual analogic scale.

^(a)^Individually calibrated stimulus pressure for the SREP procedure Kg/cm^2^.

^(b)^Median (interquartile range) values instead of mean ± SD for TSP-related variables (non-normal distribution).

Furthermore, as this is the first study in which the SREP protocol was applied to male participants, it is warranted to report SREP values separately by sex. This descriptive reporting may offer initial insights into whether sex-related differences could explain minor discrepancies in SREP responses when compared to previous studies conducted exclusively with female samples^21^.

In accordance with objective 1 and the associated hypothesis 1, based on the repeated nature of the dynamic evoked pain measures, a repeated-measures ANOVA with one between-subjects factor (Group: CLBP vs. healthy) and one repeated-measures factor (the pain ratings of the SREP or TSP protocol) was used to examine the pain response patterns for SREP and TSP. Additionally, the non-parametric Friedman test was used for the same purpose in the case of TSP to corroborate the outcomes of this non-normally distributed variable. To rule out possible confounders, repeated-measures ANOVA analyses were conducted, jointly including covariates that could potentially affect both SREP and TSP. The literature on factors influencing pain perception particularly highlights the relevance of sex in the case of TSP^57^ (with an unknown effect on SREP)^21^, and pain catastrophizing in the case of SREP (which may also influence TSP)^21,58^. Although participants were asked not to take analgesic medication in the 24 h prior to the study, the large proportion of patients with CLBP who regularly used different types of such medication (72.5%) must be taken into account. Both chronic consumption and the requested cessation for a day could have potentially affected pain perception and dynamics. Consequently, given the per-group sample size (n = 40), we limited the adjusted repeated-measures ANOVA models to a maximum of three covariates in order to preserve statistical power and model stability. This decision follows a principle of parsimony and avoids overfitting, ensuring stable parameter estimates with the available sample size. Accordingly, in all these analyses, the independent variables were Group (between-subjects) and Trial/Rating (within-subjects), with the dependent variable being the trial-wise pain ratings from the SREP or TSP protocols. In the adjusted repeated-measures ANOVAs, sex, pain catastrophizing, and regular analgesic use were included as covariates. The Greenhouse–Geisser correction was applied to adjust the degrees of freedom, and results are reported with the original degrees of freedom and the corrected *p* values.

Effect sizes were estimated using adjusted eta-squared (η^2^), Cramers´ *V* (φ_*c*_ for dichotomous variables), or the probability of superiority (PS, for TSP-related variables).

Following objective 2 and its stated hypothesis 2, Pearson correlations (Spearman for TSP-related variables) were conducted to examine the associations of SREP and TSP with sociodemographic, clinical, and evoked pain measures. In addition, subgroup analyses compared patients diagnosed with CLBP who had low (< 1) SREP scores to those whose scores were high (≥ 1) to explore potential links between SREP and clinical or psychological factors. In these analyses, SREP subgroup was the independent variable, and sociodemographic, clinical, and TSP-related measures were the dependent variables. This approach also allowed us to evaluate whether SREP responses might be accounted for by clinical or psychosocial characteristics (complementing objective 1 and hypothesis 1).

Finally, building on objective 3 and hypothesis 3, to discriminate between CLBP and healthy participants, logistic regression analyses determined the sensitivity, specificity, and overall discrimination accuracy of the pain threshold + tolerance, SREP, and TSP measures, setting Group (CLBP or healthy) as the dependent variable and pain threshold, pain tolerance, the SREP index, and the TSP index as predictors. The sensitivity index shows the proportion of patients with CLBP correctly detected (true positives vs. false negatives), whereas specificity refers to the proportion of healthy individuals correctly classified (true negatives vs. false positives).

Additionally, receiver operating characteristic (ROC) analyses were performed to determine the most accurate cut-off point for discriminating between patients with CLBP and healthy individuals using the SREP index. Thus, the SREP index was entered as the “Test variable” (predictor), and Group as the “State variable” (outcome), in the ROC curve procedure, selecting “Coordinate points of the ROC curve”. The cut-off point was estimated using the best combination, determined based on (I) the highest Youden index (Youden’s J statistic = Sensitivity + Specificity—1), and (II) sensitivity and specificity greater than 60%. The area under the ROC curve (AUC) was estimated to obtain another indicator of discrimination accuracy for the SREP index. Given that all analyses were conducted to test specific hypotheses with different variables (and not alternative hypothesis), no adjustments for repeated statistical testing were made, as is recommended in these cases^59^.

## Results

### Group comparisons—sample characteristics

**Fig. 1:**
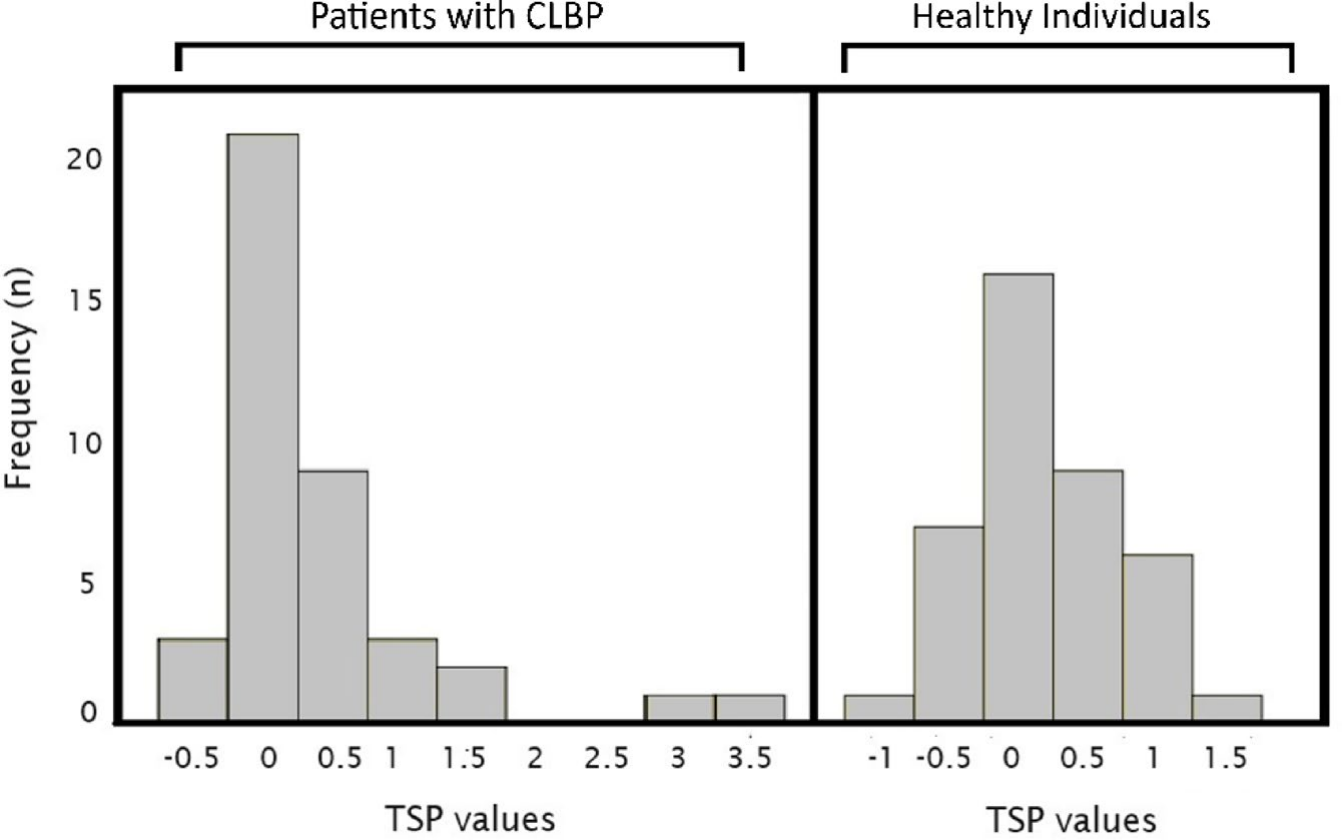
Frequency histogram of temporal summation of pain (TSP) values in patients with CLBP (left) and healthy participants (right). CLBP = Chronic Low Back Pain; TSP = difference between pain ratings (assessed by a 0–10 numeric rating scale) for the last and first stimuli of TSP protocol (averaged values of 2 series).

As can be observed in Table 1, patients with CLBP displayed higher values in all clinical variables and the SREP index. Nevertheless, no differences were observed in pain threshold, pain tolerance, or the first pain rating for SREP. Regarding the non-normally distributed TSP-related variables, neither TSP nor the first TSP pain rating showed significant group differences. In addition to Table 1, Fig. 1 shows a frequency histogram of the TSP values of patients with CLBP (right) and healthy participants (left), in order to facilitate understanding of the TSP response in each group. The majority of patients with CLBP showed regular use of medication, especially analgesics and anxiolytics, whereas only two healthy participants used some type of medication regularly. Regarding the clinical severity of the patients, the clinical pain score (MPQ) was 27.68 ± 8.20, and the CS-related symptoms score (CSI) was 36.50 ± 18.48; 15 patients (37.5%) showed symptoms with a significant level of CS (i.e., a CSI score > 40).

In examining SREP values by sex, no significant difference (t = −0.69; *p* = 0.49) in sensitization was observed between female (x̅ = 0.69 ± 1.02) and male (x̅ = 0.93 ± 1.11) patients with CLBP. Among healthy participants, there was a non-significant difference was found (t = 0.98; *p* = 0.34) either. However, whereas healthy women showed minimal habituation (x̅ = –0.27 ± 1.25), in line with previous SREP studies reporting values near zero^21^, healthy men tended to display a somewhat greater deviation from zero (x̅ = –0.61 ± 0.94), a pattern not previously explored in SREP studies.

### SREP versus TSP group responses—objective/hypothesis 1

**Fig. 2:**
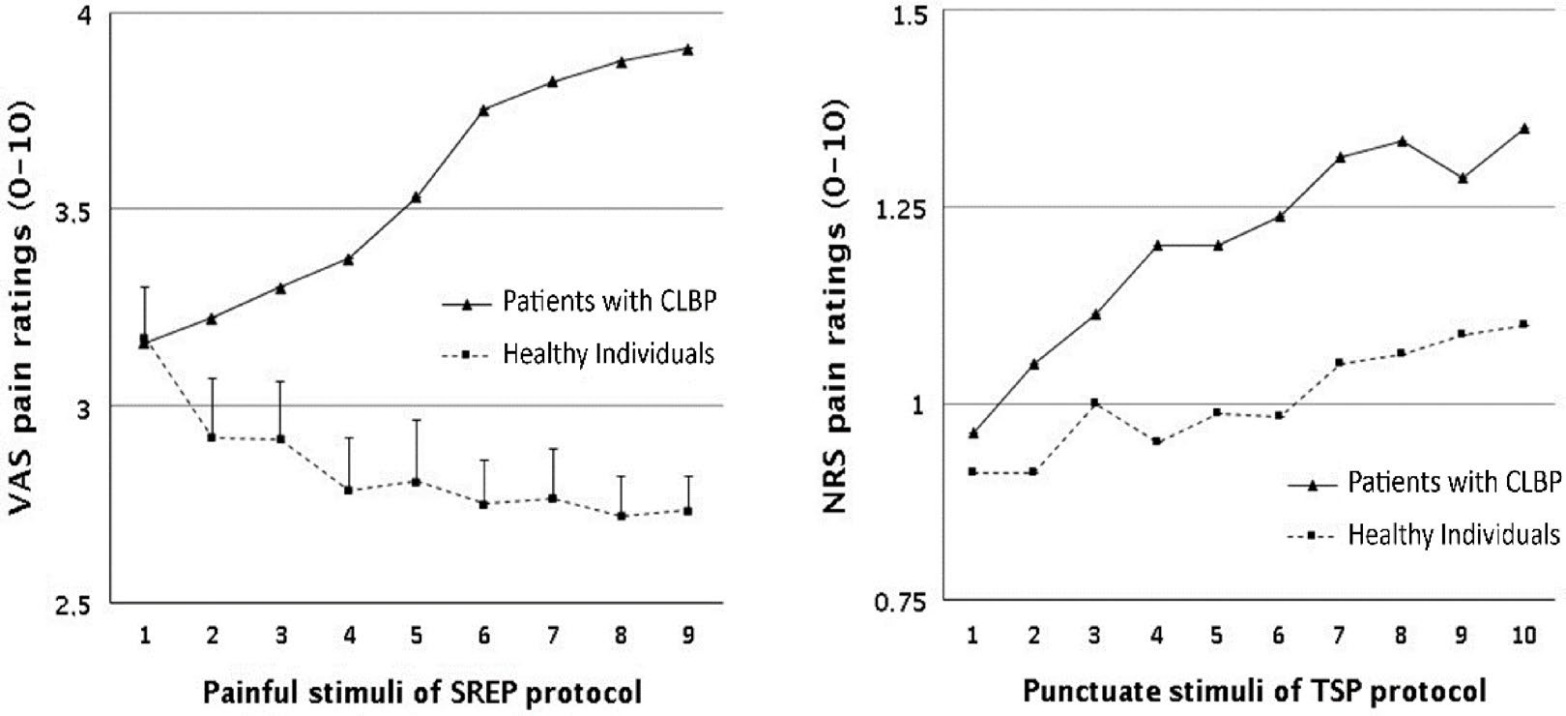
Pain ratings of patients with chronic low back pain (CLBP) and healthy individuals across the slowly repeated evoked pain (SREP) and temporal summation of pain (TSP) protocols. CLBP = Chronic Low Back Pain. (Left) Mean and SE of evoked pain ratings (0–10 visual analogic scale scores) over repeated stimuli as a function of group across the 9 painful trials of SREP protocol. (Right) Mean and SE of evoked pain ratings (0–10 numeric rating scale scores) over repeated stimuli as a function of group across the 10 punctuate stimuli (average of 2 series).

Figure 2 displays the patterns of pain ratings during the SREP (left) and TSP (right) protocols. Pain intensity ratings increased across the nine SREP stimuli [main effect of trials: F(8624) = 3.18, *p* = 0.045, η^2^ = 0.04] as a function of group [group × trial interaction: F(8624) = 14.66, *p* < 0.001, η^2^ = 0.16]. While pain ratings increased progressively in patients with CLBP [F(8312) = 14.24, *p* < 0.001, η^2^ = 0.27], they decreased in healthy participants [F(8312) = 3.47, *p* = 0.036, η^2^ = 0.08]. There were no group differences in overall perceived pain intensity between the CLBP and healthy groups [between-subjects main effect of group: F(178) = 2.73, *p* = 0.103, η^2^ = 0.03, 95% CI [−0.14, 1.56] for the mean difference].

To rule out the potential confounders that might alter the SREP response, primary analysis was repeated including sex, pain catastrophizing and regular analgesic use as covariates. While the group × trial interaction remained significant [F(8600) = 4.71, *p* = 0.011, η^2^ = 0.06], the group × covariate interactions were non-significant: sex [F(8600) = 0.89, *p* = 0.41, η^2^ = 0.01], catastrophizing [F(8600) = 0.34, *p* = 0.71, η^2^ < 0.01], and regular analgesic use [F(8600) = 0.35, *p* = 0.70, η^2^ = 0.01].

Regarding TSP, the pain ratings increased across the 10 punctuate stimuli of the TSP protocol [main effect of trials: F(9702) = 7.73, *p* < 0.001, η^2^ = 0.09] regardless of group [group × trial interaction: F(9702) = 1.27, *p* = 0.29, η^2^ = 0.02]. This was corroborated by the non-parametric analysis, confirming that pain ratings increased significantly across the 10 punctuate stimuli of the TSP protocol [χ^2^(9) = 51.92, *p* < 0.001]; this increase was significant in both patients with CLBP [χ^2^(9) = 50.62, *p* < 0.001] and healthy participants [χ^2^(9) = 18.97, *p* = 0.025].

When the primary analysis was repeated to include the three covariates, the group × trial interaction remained non-significant [F(9675) = 1.35, *p* = 0.257, η^2^ = 0.02]. Similarly, the group × covariate interactions were non-significant for sex [F(9675) = 2.05, *p* = 0.103, η^2^ = 0.03], and regular analgesic use [F(9675) = 0.63, *p* = 0.61, η^2^ = 0.01]. However, the interaction was significant for pain catastrophizing [F(9675) = 9.39, *p* < 0.001, η^2^ = 0.11], indicating that greater catastrophizing was associated with higher pain ratings during the TSP (see below).

### Associations of SREP/TSP with clinical and evoked pain factors in CLBP—Obj./Hip. 2

As Table 2 shows, while there was no significant correlation between SREP and any other variable in patients with CLBP, TSP correlated positively with BMI, pain catastrophizing, current clinical pain, clinical pain, and CS-related symptom severity, and negatively with pain threshold and tolerance. The SREP and TSP indexes did not correlate with each other (*ρ* = −0.14, *p* = 0.37). Furthermore, the TSP index did not correlate with the first TSP pain rating (*ρ* = 0.15, *p* = 0.370), and the SREP index did not correlate with the first SREP pain rating (r = 0.03, *p* = 0.870).

**Table 2:** Correlations between SREP (Pearson) and TSP (Spearman) and sociodemographic, clinical and evoked pain variables in chronic low back pain (CLBP) participants.

Variables	Patients with CLBP (n = 40)	
	SREP	TSP
	*r; p*	*ρ; p*
Age (years)	0.018; 0.913	0.104; 0.523
Body mass index	0.130; 0.425	0.317; 0.046
Chronicity (years)	− 0.334; 0.085	< 0.001; 0.999
Catastrophizing (CSQ-Cat)	0.085; 0.829	0.454; 0.003
State anxiety (STAI-S)	− 0.035; 0.833	0.156; 0.335
Sleep satisfaction (OSQ-Sat)	0.006; 0.973	− 0.053; 0.744
Anxiety (HADS-A)	0.035; 0.828	0.249; 0.121
Depression (HADS-D)	0.131; 0.421	0.262; 0.102
Current pain intensity (0–10 VAS)	0.079; 0.628	0.362; 0.022
Clinical pain (MPQ)	0.035; 0.829	0.314; 0.049
Central sensitization (CSI)	− 0.101; 0.536	0.316; 0.047
Pain threshold (Kg)	0.265; 0.098	− 0.495; 0.001
Pain tolerance (Kg)	0.108; 0.508	− 0.415; 0.008

Abbreviations. CSI = Central Sensitization Inventory; CSQ-Cat = pain catastrophizing subscale of Coping Strategies Questionnaire; HADS = Hospital Anxiety (A) and Depression (D) Scale; MPQ = McGill Pain Questionnaire; NA = not applicable; NRS = numeric rating scale; OSQ-Sat = satisfaction subscale of Oviedo Sleep Questionnaire; SREP = slowly repeated evoked pain sensitization; STAI-S = state subscale of State-Trait Anxiety Inventory; TSP = temporal summation of pain response; VAS = visual analogic scale.

*r* = Pearson correlation coefficient; *ρ* = Spearman correlation coefficient.

There were no significant differences (all ps ≥ 0.24) between patients with CLBP presenting lower versus higher SREP scores (< 1 vs. ≥ 1).

### Discrimination accuracy of evoked pain measures—Obj./Hip. 3

**Fig. 3:**
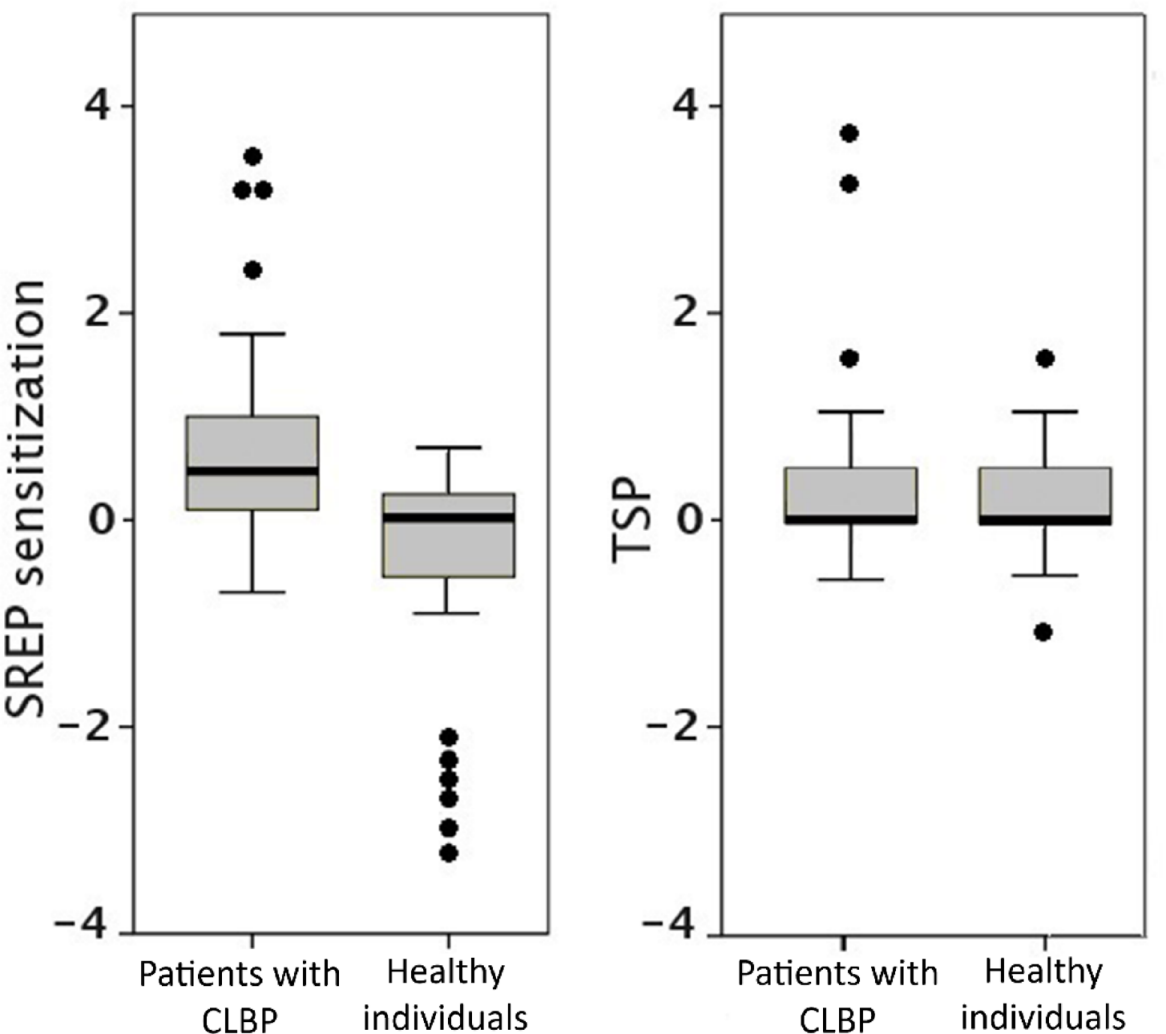
Between-groups overlap for slowly repeated evoked pain (SREP) sensitization (left) and temporal summation of pain (TSP) (right). CLBP = Chronic low back pain; TSP = difference between pain ratings (assessed by a 0–10 numeric rating scale) for the last and first stimuli of TSP protocol (averaged values of 2 series); SREP sensitization = difference between pain ratings (assessed by a 0–10 visual analogic scale) for the last and first stimuli of SREP protocol.

Logistic regressions showed that SREP sensitization was the only variable that discriminated between patients with CLBP and healthy participants (β =  − 1.86, SE = 0.57, 95% CI [−2.98, −0.74], Wald = 10.66, *p* = 0.001; adjusted R^2^ = 0.39). The other evoked pain measures did not allow for significant discrimination, i.e., pain threshold + tolerance (β = 0.06, SE = 0.14, 95% CI [−0.21, 0.33], Wald = 0.18, *p* = 0.671, for threshold; β =  − 0.05, SE = 0.11, 95% CI [−0.27, 0.17], Wald = 0.23, *p* = 0.630, for tolerance; adjusted R^2^ = 0.01) and TSP (β =  − 0.42, SE = 0.35, 95% CI [−1.11, 0.27], Wald = 1.389, *p* = 0.239; adjusted R^2^ = 0.03). Table 3 displays the discriminative accuracy in addition to the sensitivity and specificity values. To facilitate comparison of the overlap in between-group distributions of dynamic pain indicators, Fig. 3 shows box-and-whisker plots based on the logistic regression models of SREP and TSP as a function of group. The overlap in distributions was smaller for SREP than for TSP. Two patients with CLBP (5%) had lower SREP sensitization values than the mean value of the healthy group, while no healthy participants had a SREP sensitization value greater than the CLBP mean. However, 16 patients with CLBP (40%) had lower TSP values than the mean value of the healthy group (the same result was obtained when using the median value), and 25 healthy participants (62.5%) had a greater TSP value than the CLBP mean (4 healthy participants [10%] when using the median value).

**Table 3:** Sensitivity and specificity values and overall accuracy of pain threshold + tolerance (combined), slowly repeated evoked pain (SREP) and temporal summation of pain (TSP) for discriminating between chronic low back pain patients and healthy participants.

	Threshold + Tolerance	SREP sensitization	TSP
Sensitivity	0.53	0.70	0.40
Specificity	0.53	0.63	0.58
Overall accuracy	52.5%	66.3%	49%

### Cut off for SREP sensitization—Obj./Hip. 3

According to the highest Youden index (J = 0.38) derived from ROC analyses of SREP sensitization (AUC = 0.79, SE = 0.05, *p* < 0.001, 95% CI = 0.68–0.88), and the sensitivity and specificity criterion of ≥ 0.60, the SREP value identified as the most sensitive cut-off point to discriminate between patients with CLBP and healthy participants was 0.05.

Considering this SREP sensitization cut off (0.05), and taking into account the fact that SREP values offer only one decimal (i.e., the 0–10-point VAS used for rating pain stimuli offers a maximum resolution of 1 decimal), the use of this cut-off in clinical practice allows a 77.5% of probability of successful detection of patients with CLBP when SREP is positive (i.e., ≥ 0.1), whereas it can detect healthy status with 60% probability when SREP is null or negative (i.e., ≤ 0).

## Discussion

As anticipated, patients with CLBP in our sample presented a less favorable clinical-psychological profile than healthy participants, with poorer sleep satisfaction, higher current and chronic pain intensity, greater pain catastrophizing, and more pronounced symptoms of anxiety and depressed mood. The more frequent regular use of analgesics (both non-opioid and opioid), anxiolytics, and antidepressants in this group further reflected the multidimensional nature and complexity of their condition. These baseline characteristics are in line with previous studies assessing similar variables in CLBP populations^28,60^ and support the representativeness of our cohort.

Aligned with Objective/Hypothesis 1, we characterized dynamic pain responses using SREP and TSP to differentiate CLBP from healthy status. While pain threshold, pain tolerance, and TSP did not differ between groups, SREP sensitization was evident in patients with CLBP and blunted or absent in healthy individuals. Patients exhibited a clear, progressive increase in SREP pain ratings, whereas healthy participants showed slight habituation; importantly, these group differences were not attributable to initial pain ratings or to overall perceived intensity across stimuli, indicating a genuine divergence in dynamic modulation. By contrast, TSP increased significantly in both groups with comparable rates, failing to show a group effect, less conclusive than reports suggesting heightened TSP in CLBP^18,19^ and consistent with the mixed findings recently noted^24^. Overall, Hypothesis 1 was partially supported: the predicted SREP sensitization pattern in CLBP (and its absence in healthy controls) was confirmed (consistent with prior SREP findings in CS-related conditions)^34–36^, whereas the expected between-group difference in TSP was not. Additionally, the SREP response remained stable when the most relevant clinical and psychological variables were introduced as covariates in the analyses, further supporting the notion that these factors did not influence the response. This interpretation is reinforced by the fact that stratifying patients by SREP magnitude (SREP < 1 vs. SREP ≥ 1) revealed no significant differences in the measured psychosocial or clinical variables, which is consistent with the lack of association observed in relation to the following objective.

Regarding Objective/Hypothesis 2**,** no clinical or psychological factor was associated with SREP sensitization in this study. These results are contrary to previous SREP studies in fibromyalgia and episodic migraine patients, in which pain catastrophizing, levels of clinical pain, or sleep problems were associated with SREP index^33–36^. In contrast, in patients with CLBP, TSP was positively associated with current and usual clinical pain, CS-related symptoms, and pain catastrophizing. Previous studies support these associations with TSP in patients with CLBP^17,30,32^ and even in healthy individuals^26,27^. In addition, TSP was also positively associated with BMI. Overweight is known to be related to chronic systemic inflammation, which can promote nociception via the production of pro-inflammatory cytokines by adipose tissue, leading to sensitization of peripheral nociceptors and nociceptive transmission pathways^61,62^. Regarding the associations with pain threshold and tolerance, TSP was related to these static evoked pain measures in this study, whereas SREP was not. Thus, TSP appears to be a more predictive index of the severity of clinical symptoms, the level of general pain sensitivity (the lower the threshold and tolerance, the greater the TSP), and some physical characteristics, such as a high BMI, in patients with CLBP. In this regard, SREP was not useful to predict the severity of the symptomatology that usually accompanies CLBP.

In relation to Objective/Hypothesis 3, the SREP index demonstrated a discrimination accuracy of 66.3% for distinguishing patients with CLBP from healthy individuals. In contrast, the detection accuracy for TSP was equivalent to chance (50%), and static pain measures (pain threshold and tolerance) achieved 53%. Thus, SREP showed significantly greater discriminative capacity than both TSP and static measures, although its overall accuracy is still insufficient to consider it a reliable standalone marker for CLBP. The most useful cut-off point for differentiating CLBP from healthy status corresponded to a SREP value of 0.05. Using a threshold of SREP ≥ 0.1 (positive SREP) allowed detection of patients with CLBP with 77.5% accuracy, whereas applying a threshold of SREP ≤ 0 (null or negative SREP) identified healthy individuals with 60% accuracy.

In sum, while SREP appears to be preferable for characterizing pain responsiveness and for discriminating patients with CLBP from healthy individuals, with a discrimination accuracy exceeding 66%, TSP seems to be a better indicator of the severity of CLBP-associated symptoms. Unlike findings in fibromyalgia or episodic migraine^34–36^, the SREP index does not seem to be useful for assessing the severity of CLBP symptomatology. Conversely, TSP (primarily explained by spinal wind-up effects) showed clear limitations in distinguishing CLBP from healthy status. The observation that SREP can differentiate groups both qualitatively and quantitatively (pain increases in CLBP versus slight habituation in healthy participants) suggests that: (i) SREP may provide a broader assessment of CS processes, not restricted to wind-up mechanisms, which cannot be elicited under the slow stimulation frequencies used in TSP^15^; and (ii) in some patients with CLBP, the development of CS may be better explained by multiple CS mechanisms, beyond spinal wind-up, that would be scarcely present in healthy individuals. A step forward in relation to the present study would be to conduct additional research recruiting patients with CLBP with centralized pain versus nociceptive profiles^7–9^. For example, selecting a clear patient profile through clinical and sensory evaluation by experts in the management and treatment of CLBP could help to define the real scope of SREP as an index specifically detecting CS processes in this chronic pain condition. Taken together, the present findings not only highlight this potential, but also justify the design of future targeted studies aimed at confirming whether SREP can serve as a clinically useful tool for identifying those patients with CLBP whose pain presentation involves a CS component, thereby refining diagnosis and guiding more tailored interventions.

Supporting this interpretation, SREP and TSP were not correlated in the present study. A similar lack of association has been reported in rheumatoid arthritis, whereas a significant correlation was observed in fibromyalgia. This might reflect the greater magnitude of CS in fibromyalgia, where SREP and TSP show elevated responses that allow them to be associated^37^. Whether SREP sensitization can serve as a general marker of CS should be confirmed in future research, ideally by comparing SREP with other indicators such as CPM, which is specifically sensitive to descending inhibitory pain pathways^16^.

From a mechanistic standpoint, SREP is elicited at a stimulation frequency roughly ten times lower than that used for TSP^21,37^, making it improbable that both rely on the same underlying process. Previous evidence suggests that SREP, unlike TSP, selectively captures sensitization in CS-related pain populations and is absent in healthy individuals or pain diseases more related to peripheral etiology, such as rheumatoid arthitis^37^. One plausible explanation is that descending inhibitory control from higher brain centers more effectively suppresses generalized CS in healthy subjects during physiological noxious stimulation. Accordingly, a working hypothesis is that the physiological basis of SREP may involve deficient inhibition of CS processes such as long-term potentiation at the spinal cord cellular level, a mechanism known to amplify pain and mediate neurogenic hyperalgesia in chronic pain conditions^63–65^. Clarifying these mechanisms remains a key target for future research and partially limits the scope of the present conclusions.

Although the sample size can be considered sufficient to meet the primary aims, the statistical power for sex-based comparisons was limited. Notably, unlike in previous SREP studies, healthy participants in the current sample showed a greater degree of habituation during SREP, potentially explained by lower pain responsiveness in males (x̅ = –0.61) than in females (x̅ = –0.27). This pattern is consistent with prior evidence of sex differences in evoked pain indicators^17,66^ and with earlier SREP outcomes reported in healthy females^33–36^. Replicating the study with a larger and more balanced sample would allow for robust sex-specific analyses. Nevertheless, the significant differences in pain responses between CLBP and healthy groups observed here with the SREP index were independent of sex, pain catastrophizing, and regular medication use. Another limitation was that, although the condition order (CLBP or healthy) was counterbalanced, the investigator was not blinded.

Future research should investigate SREP in CS pain conditions other than fibromyalgia and episodic migraine (e.g., temporomandibular disorder or irritable bowel syndrome) and in partly centralized pain conditions, given its proven ability to discriminate pain sensitization in sickle cell disease^67^. Building on this, it would also be valuable to examine SREP performance in CLBP populations specifically classified by their pain profile, as this could refine understanding of its applicability for detecting CS in clinical practice.

Third, certain methodological limitations should be acknowledged: (a) the use of different pain rating scales and stimulation modalities (VAS and pressure in SREP vs NRS and pinprick in TSP), which, although determined by protocol-specific constraints, may affect the comparability of responses; and (b) the use of a protocol with stronger evidence for reliably detecting TSP in CLBP would have strengthened the study. These methodological considerations should be taken into account in future studies.

Beyond these points, further SREP research should compare its outcomes with dynamic pain indicators other than TSP (e.g., CPM), and incorporate longitudinal designs to assess the long-term predictive power of SREP for CLBP development and maintenance. Additionally, building on earlier findings linking SREP sensitization to objective markers of sympathetic activity such as skin conductance^68^, there remains a need to examine its relationship with objective CS markers like transcranial Doppler ultrasonography, which offers high temporal resolution. Clarifying the mechanisms underlying SREP sensitization remains a key target for future research and partially limits the scope of the present conclusions.

In this study, SREP was implemented as part of a diagnostic-test design (not with the aim of diagnosing CLBP), aimed at characterizing pain responsiveness in CLBP, examining its relationship with clinical and psychological factors typical of these patients, and assessing its ability to discriminate between groups. This constitutes an initial step toward determining its potential clinical utility. The findings support the need for future research to confirm whether SREP can, in practice, contribute to identifying patients with CLBP with a centralized pain profile or a predisposition to heightened pain sensitization. In contrast, TSP demonstrated predictive value for clinical severity but lacked discriminatory capacity, highlighting a complementary role for both protocols in patient assessment.

This work should be regarded as a preliminary stage that supports conducting studies in stratified CLBP populations to validate SREP’s role in profiling pain sensitization, refining prognosis, and guiding personalized treatment strategies. By enabling earlier recognition of CS, SREP could (once validated) facilitate more targeted interventions such as antidepressants, cognitive-behavioral therapy, or tailored physical therapies in patients most likely to benefit^69^.

## Data Availability

The datasets generated during and/or analyzed during the current study are available from the corresponding author on reasonable request.
